# Pathological Changes of Small Vessel Disease in Intracerebral Hemorrhage: a Systematic Review and Meta-analysis

**DOI:** 10.1007/s12975-023-01154-4

**Published:** 2023-06-06

**Authors:** Mangmang Xu, Yuyi Zhu, Xindi Song, Xuelian Zhong, Xinxin Yu, Deren Wang, Yajun Cheng, Wendan Tao, Bo Wu, Ming Liu

**Affiliations:** 1 Department of Neurology, West China Hospital, Sichuan University, No. 37 Guo Xue Xiang, Chengdu, 610041 Sichuan Province China; 2 West China School of Nursing, Sichuan University/West China Hospital of Sichuan University, Chengdu, Sichuan Province China; 3 Department of Orthodontics, ChengDu Dental Hospital, Chengdu, Sichuan Province China; 4 Center of Cerebrovascular Diseases, West China Hospital, Sichuan University, Sichuan Province Chengdu, China

**Keywords:** Intracerebral hemorrhage, Pathology, Arteriolosclerosis, Cerebral amyloid angiopathy, Meta-analysis

## Abstract

In intracerebral hemorrhage (ICH) with pathology-proven etiology, we performed a systematic review and meta-analysis to elucidate the association between cerebral amyloid angiopathy (CAA) and arteriolosclerosis, and directly compared MRI and pathological changes of markers of cerebral small vessel disease (CSVD). Studies enrolling primary ICH who had received an etiological diagnosis through biopsy or autopsy were searched using Ovid MEDLINE, PubMed, and Web of Science from inception to June 8, 2022. We extracted pathological changes of CSVD for each patient whenever available. Patients were grouped into CAA + arteriolosclerosis, strict CAA, and strict arteriolosclerosis subgroups. Of 4155 studies identified, 28 studies with 456 ICH patients were included. The frequency of lobar ICH (*p*<0.001) and total microbleed number (*p*=0.015) differed among patients with CAA + arteriolosclerosis, strict CAA, and strict arteriolosclerosis. Concerning pathology, severe CAA was associated with arteriolosclerosis (OR 6.067, 95% CI 1.107–33.238, *p*=0.038), although this association was not statistically significant after adjusting for age and sex. Additionally, the total microbleed number (median 15 vs. 0, *p*=0.006) was higher in ICH patients with CAA evidence than those without CAA. The pathology of CSVD imaging markers was mostly investigated in CAA-ICH. There was inconsistency concerning CAA severity surrounding microbleeds. Small diffusion-weighted imaging lesions could be matched to acute microinfarct histopathologically. Studies that directly correlated MRI and pathology of lacunes, enlarged perivascular spaces, and atrophy were scarce. Arteriolosclerosis might be associated with severe CAA. The pathological changes of CSVD markers by ICH etiology are needed to be investigated further.

**Supplementary Information:**

The online version contains supplementary material available at 10.1007/s12975-023-01154-4.

## Introduction

Primary intracerebral hemorrhage (ICH) results in high morbidity and disability. Cerebral small vessel disease (CSVD) is the major cause of primary ICH [1]: arteriolosclerosis, which is closely associated with hypertension [2], mainly accounts for deep ICH, and cerebral amyloid angiopathy (CAA) accounts for lobar ICH. Notably, CAA and arteriolosclerosis coexist frequently. Previous studies have found that lobar ICH/microbleed and deep ICH/microbleed could coexist in a patient [3]. Additionally, animal studies have found that an increase in systolic blood pressure promotes the occurrence of ICH in Tg2576 mice characterized by amyloid beta (Aβ) deposition [4]. And in a clinical study, subgroup analysis of the PROGRESS study has shown that antihypertensive treatment (vs. placebo) reduced the risk of ICH in CAA patients [5]. However, there is currently a lack of systematic reviews that assess the association between CAA and arteriolosclerosis in ICH patients with pathology-proven evidence.

On the other hand, increasing evidence has demonstrated that imaging markers of CSVD are related to the presence and prognosis of primary ICH [6, 7]. However, the characteristics of CSVD markers in patients with pathological evidence for arteriolosclerosis- and CAA-related ICH, and the underlying pathological changes of theses markers, remain unclear. Although a previous study [8] reviewed the pathological substrates of CSVD such as cerebral microbleeds (CMB) and white matter changes, the majority of included studies investigated Alzheimer’s disease. Despite 80~97% of patients with Alzheimer’s disease have CAA at autopsy, most of them do not suffer from symptomatic ICH [9, 10]. We, therefore, performed a systematic review and meta-analysis to elucidate (1) the association between arteriolosclerosis and CAA and their imaging characteristics, and (2) the imaging and pathological correlations of CSVD markers in primary ICH patients to better understand the mechanisms underlying primary ICH.

## Methods

### Search Strategy

**Fig. 1:**
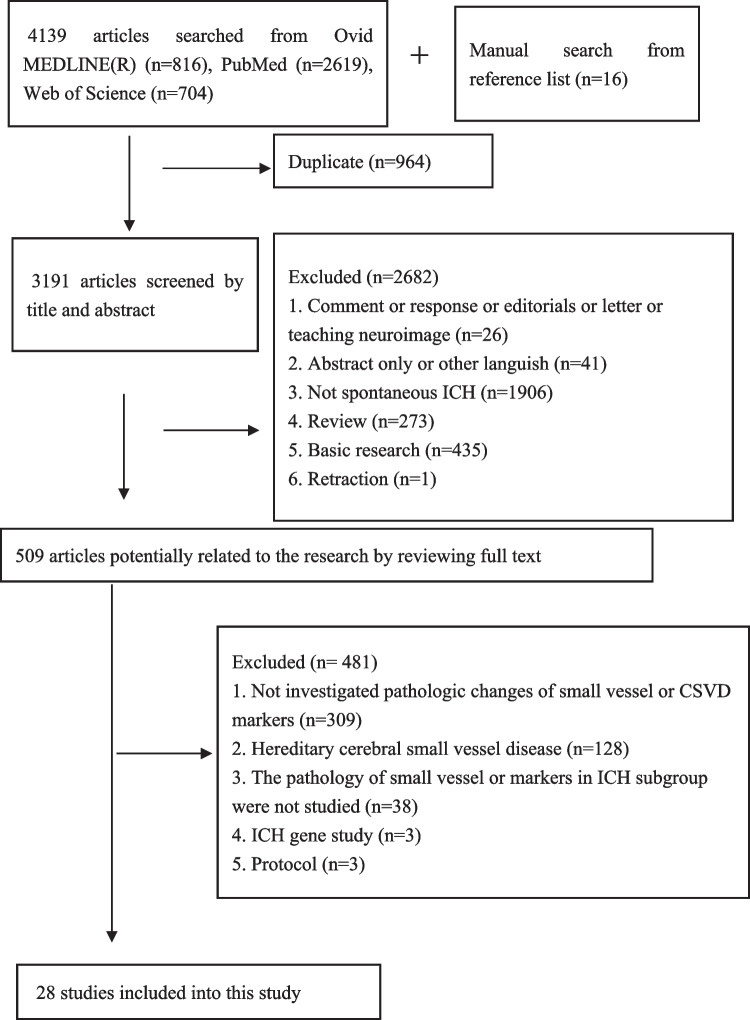
Flow chart of study selection

Our study was approved by the Ethics Committee on Biomedical Research, West China Hospital of Sichuan University, and was registered in PROSPERO (CRD42022343347). We followed the Preferred Reporting Items for Systematic Reviews and Meta-Analyses [11] guidelines. We systematically searched PubMed, Ovid Medline, and Web of Science for studies investigating the neuropathology of CSVD in patients with primary ICH until 8 June 2022. The following terms were used: “(cerebral hemorrhage) OR (intracranial hemorrhage)” AND “(patholog*) OR (postmortem) OR (autopsy) OR (biopsy)” AND “(cerebral small vessel diseases) OR (microvessel) OR (white matter) OR (microinfarct) OR (lacune) OR (perivascular space) OR (microbleed) OR (microhemorrhage) OR (atrophy).” Additionally, references to relevant studies were searched. The details of the study selection process are presented in Fig. 1.

### Eligibility Criteria

The included criteria were as follows: investigating adult patients (age≥18 years) with primary ICH; having autopsy or biopsy to confirm the etiology for arteriolosclerosis and CAA; with or without CSVD markers, including white matter changes, CMB, cerebral microinfarcts (CMI), lacunes, enlarged perivascular spaces (EPVS), brain atrophy, and the total CSVD burden. Abstract, review, editorial, and basic research were excluded. This systematic review only focused on primary ICH, not on hemorrhage into the subarachnoid space, epidural space, or subdural space. Pure intraventricular hemorrhage, ICH secondary to trauma, structural vascular lesions, inflammation, infections, inherited or genetic small vessel diseases, and hemorrhagic transformation after ischemic stroke were beyond the scope of this review. We only included studies that were published in English.

### Data Extraction

Two authors (Mangmang Xu and Xindi Song) independently searched the literature and extracted data. Any disagreement was resolved by consensus. A data extraction form was developed using SPSS. For each included study, we extracted the family name of the first author, publication year, country of origin, the sample size of primary ICH, examination method (autopsy or biopsy), patient demographics (male sex, age at death or biopsy), pathology diagnosis (CAA or arteriolosclerosis), ICH location (lobe, deep, cerebellum), and CSVD imaging markers including white matter changes, lacunes, CMB, CMI, EPVS, atrophy, and total CSVD burden. For studies that investigated ICH with CAA evidence, we additionally extracted information on the diagnostic method of CAA, CAA-related small vessel wall changes (SVWCs), the coexistence of arteriolosclerosis, senile plaques, and blood vessel changes in or around the hematoma.

### Definitions

Lobar ICH included hemorrhages originating from the frontal, temporal, parietal, or occipital lobes of the brain. For the two studies [12, 13] that enrolled clinically diagnosed CAA-ICH according to the modified Boston criteria without reporting ICH location, we regarded those cases as having lobe ICH. Deep ICH was considered when originating from the basal ganglia, thalamus, internal capsule, external capsule, or brain stem. Biology included brain biopsy and biopsy at hematoma evacuation in this study.

Severe CAA in histopathology was defined as follows: (1) a CAA score of 9–12 for the study [12] that used a cumulative cortical CAA score with a range of 0–12 by calculating the basis of Aβ stained sections from four areas, (2) a CAA score of 3–4 for those studies [14–17] that used the grading scale by Greenberg and Vonsattel with a range of 0–4, (3) a CAA score of 3+ indicating that many lesions were visible [18] in a scale of 1+~3+, or as per the original study authors’ definition [19–22].

Arteriolosclerosis was defined if there existed lipofibrohyalinosis [20], lipohyalinosis [21, 23], arteriolosclerosis [13, 18, 19, 24–28], or hypertensive vasculopathy [12, 13, 17] on pathology. For the study [21] that performed a pathological examination and concluded that there were no other possible causes of ICH except CAA, we considered those cases as strict CAA.

### Statistical Analysis

Most of the included studies were case reports, so we extracted individual patient data from those cases. The demographic data in the study by Ter Telgte [12] were the same as that in the study by van Veluw [13]; therefore, we only extracted data from the latter. For CMB analysis, we extracted CMB number in ex vivo MRI in the study by van Veluw 2016 [17], under the other two studies [13, 20]. Independent-samples *T* test, one-way ANOVA, Mann-Whitney *U* test, or Kruskal-Wallis *H* test were used for continuous variables, and Pearson chi-squared test or Fisher’s exact test for categorical variables, when appropriate. Multivariate analysis was performed using binary logistic regression. For the statistical analysis in our present study, hemorrhage in the cerebellum was categorized as lobar ICH. All statistical analyses were performed using IBM SPSS Statistics (version 23), and a *p* value of ≤0.05 was considered statistically significant.

## Results

### Study Characteristics

A total of 4155 citations and 509 full texts were screened (Fig. 1). Of these, 28 studies with 456 participants were included [12–39]. The characteristics of included studies are shown in Table 1. Most of the studies were conducted in the USA (39.3%, 11/28), followed by Japan (28.6%, 8/28). Three hundred twenty patients had CAA with or without arteriolosclerosis; 48 patients had strict CAA while 19 had CAA + arteriolosclerosis. CAA-related SVWCs were investigated in 17 studies [14–22, 24, 29–33, 38, 39], and their detailed pathological changes are summarized in the Supplementary table.

**Table 1:** Characteristics of included studies which investigating primary ICH

Study	Country	Examination method	Sample size	Age at death or biopsy, ys	Male (%)	Pathology diagnosis	ICH location	CSVD markers on brain imaging
Charidimou 2016 [36]	USA	Biopsy, autopsy *	54	Mean 71.6	36.9	CAA without information on arteriolosclerosis	Lobe	CSVD burden, white matter changes on MRI, CMB, EPVS
Cordonnier 2010 [23]	France	Autopsy	7	68–89	14.3	CAA without information on arteriolosclerosis (5), strict arteriolosclerosis (2)	Lobe (5), deep (2)	No
Doden 2016 [32]	Japan	Biopsy	31	Mean 75.3 in CAA group, mean 72.8 in non-CAA group	32.3	CAA (22), non-CAA (9), all without information on arteriolosclerosis†	Cortical-subcortical regions	CMB, white matter changes on MRI
Fazekas 1999 [20]	Austria	Autopsy	11	45–90	54.5	CAA+ arteriolosclerosis (2), strict arteriolosclerosis (9)	Lobes (7), deep (4)	CMB, white matter changes on MRI, lacune
Gilbert 1983 [33]	Canada	Autopsy	11	67–84	27.3	CAA+ arteriolosclerosis (2), strict CAA (9)	Lobe	No
Gray 1985 [30]	France	Autopsy	9‡	55–83‡	66.7‡	CAA without information on arteriolosclerosis	Lobe	White matter changes on CT
Greenberg 2009 [34]	USA	Autopsy	6**	66–87	33.3	CAA without information on arteriolosclerosis	Lobe	CMB
Guidoux 2018 [35]	France	Autopsy	81	25–91	54.3	CAA (20), non-CAA (61), all without information on arteriolosclerosis	Deep (51), lobe (28), cerebellum (2)	CMB
Hernandez-Guillamon 2012 [22]	Spain	Autopsy	5‡‡	66–90	40.0	CAA without information on arteriolosclerosis	Lobe	CMB
Ishii 1984 [19]	Japan	Autopsy	7	66–94	57.1	CAA+ arteriolosclerosis (3), strict CAA (4)	Lobe	No
Jolink 2022 [25]	The Netherlands	Autopsy	16§	44–89	43.8	CAA+ arteriolosclerosis (3), strict CAA (6), strict arteriolosclerosis (7)	Lobe (7), deep (8), cerebellum (1)	CMB, CMI
Lin 2018 [14]	Japan	Biopsy	29	61–85	48.3	CAA (24), non-CAA (5), all without information on arteriolosclerosis	Lobe	No
Mastaglia 1969 [27]	Australia	Autopsy	2	48–87	0	Arteriolosclerosis without information on CAA	Deep	No
Oide 2003 [18]	Japan	Biopsy (52), autopsy (12)	64	61–91	42.2	CAA+ arteriolosclerosis (2), strict CAA (10), CAA without information on arteriolosclerosis (52)	Lobe (63), cerebellum (1)	White matter changes on CT
Pasi 2020 [26]	USA	Biopsy (2), autopsy (8)	10§§	31–57	70.0	Arteriolosclerosis (9), non-arteriolosclerosis (1), all without information on CAA	Lobe (2), deep (8)	CSVD burden
Pasi 2019 [37]	USA	Biopsy (5), autopsy (31)	36	Mean 70.1	54.0	CAA (26), non-CAA (10), all without information on arteriolosclerosis	Unclear	CMB
Poyuran 2019 [15]	India	Biopsy	6††	56–70	66.7	CAA without information on arteriolosclerosis	Lobe (5), deep (1)	CMB, White matter changes on CT and MRI, Atrophy on MRI
Schrag 2010 [16]	USA	Autopsy	1||||	74	0	CAA without information on arteriolosclerosis	Lobe	CMB
Shelton 2015 [31]	USA	Autopsy	1	68	0	CAA without information on arteriolosclerosis	Lobe	CMB
Takebayashi 1983 [28]	Japan	Autopsy (10), biopsy (11)	21||	37–74	76.2	Arteriolosclerosis without information on CAA	Unclear	No
Takeda 2012 [29]	Japan	Autopsy	1	86	100	CAA without information on arteriolosclerosis	Lobe	No
Takeda 2018 [24]	Japan	Autopsy	1	74	0	CAA + arteriolosclerosis	Lobe	No
Ter Telgte 2020 [12]	USA	Autopsy	6##	64–88	50	CAA + arteriolosclerosis (2), strict CAA (4)	Lobe	CMI
van Etten 2014 [38]	USA	Autopsy	9	Unclear	Unclear	CAA without information on arteriolosclerosis	Unclear	No
van Veluw 2016 [17]	USA	Autopsy	1***	87	100	CAA + arteriolosclerosis	Lobe	CMB, CMI
van Veluw 2019 [13]	USA	Autopsy	6##	64–88	50	CAA + arteriolosclerosis (2), strict CAA (4)	Lobe	CMB, CMI
Vonsattel 1991 [21]	USA	Biopsy, autopsy*	12#	57–83	66.7	CAA+ arteriolosclerosis (1), strict CAA (11)	Lobe	No
Yoshimura 1992 [39]	Japan	Autopsy	12	71–94	41.7	CAA without information on arteriolosclerosis	Lobe	No

*The number of biopsy or autopsy was unclear

^†^Nine of 22 patients in this study had primary ICH

^‡^Nine of 12 patients in this study had ICH. The age and male proportion were derived from the 12 patients

^§^Sixteen of 20 patients in this study had primary ICH with pathology-based etiology

^||^The original study included 20 patients at surgery and 16 brains at autopsy, but 21 in total went to the investigation of small vessel walls

#The original study included 17 CAA-ICH, among which 12 were sporadic

**Six of 46 patients had primary ICH with pathology evidence

^††^Six of 7 patients in this study had primary ICH.

‡‡ Five of 6 patients in this study had primary ICH

^§§^Ten of 12 patients in this study had ICH

^||||^One of 8 patients in this study had primary ICH

##Six of 12 patients in this study had primary ICH. CAA-ICHs in the two studies overlapped

***One of 5 patients in this study had ICH

### The Association Between Arteriolosclerosis and CAA

Ten studies [13, 17–21, 23–25, 33] with 79 cases assessed both CAA and arteriolosclerosis histopathologically. CAA + arteriolosclerosis accounted for 21.5% (17/79) of cases. The frequency of lobar ICH differed among CAA + arteriolosclerosis, strict CAA, and strict arteriolosclerosis (82.4%, 100%, 38.9%, respectively; *p*<0.001), so did the total number of CMB (median 7, 161, 0, respectively; *p*=0.015).

Ten studies [13–22] with 76 cases assessed the presence and severity of CAA on pathology. Data showed that severe SVWCs due to CAA were highly prevalent (67.6%, 53/76), which was found to be associated with arteriolosclerosis when compared with CAA without severe SVWCs (58.3 vs. 18.8%, *p*=0.050; OR 6.067, 95% CI 1.107–33.238, *p*=0.038). However, this association was not significant after correcting for age and sex (adjusted OR 4.959, 95% CI 0.734–33.521, *p*=0.101).

### The Clinical and Imaging Characteristics of Arteriolosclerosis and CAA

As shown in Table 2, all cases of ICH due to strict CAA aged > 55 years and had lobar ICH. CAA presence was associated with a higher number of CMB (CAA vs. non-CAA: 15 (2–104) vs. 0 (0–3), *p*=0.006, calculated from 7 studies [13, 16, 17, 20, 25, 31, 34] with 29 participants), as well as a higher number of CMI (CAA vs. non-CAA: 29 (3.5–56.25) vs. 0 (0–0), *p*=0.064, calculated from 3 studies [13, 17, 25] with 10 participants), although without reaching a statistically significant difference for the latter. And patients with strict CAA were more likely to have a higher number of total CMB (OR 1.016, 95% CI 1.002–1.030, *p*=0.029, as determined by binary logistic regression) than those with strict arteriolosclerosis or CAA+ arteriolosclerosis; this association remained significant after adjusting for age and sex (adjusted OR 1.015, 95% CI 1.001–1.029, *p*=0.040). There existed conflicting results regarding the association between ICH etiology and CMB number by location [20, 32]. For cerebellar CMB, CAA-ICH had more superficial cerebellar CMB, while non-CAA-ICH had more deep/mixed cerebellar CMB [37].

**Table 2:** The clinical characteristics by CAA severity and ICH etiology

Variables	ICH etiology (*n*=79)				The CAA severity in CAA-ICH (*n*=76)		
	CAA+ arteriolosclerosis (*n*=17)	Strict CAA (*n*=44)	Strict arteriolosclerosis (*n*=18)	*P*	Non-severe (*n*=23)	Severe (*n*=53)	*P*
Age, mean (SD)	77.2 (9.0)	75.8 (7.6)	69.9 (16.1)	0.080	74.2 (7.9)	75.1 (8.4)	0.685
Male sex, *n* (%)	12 (70.6)	18 (40.9)	7 (38.9)	0.085	12 (52.2)	26 (49.1)	0.803
Hypertension, *n* (%)	5 (41.7) §	11 (36.7) §	10 (62.5) §	0.237	8 (44.4) *	19 (38.8) *	0.675
Lobar ICH, *n* (%)	14 (82.4)	44 (100)	7 (38.9)	<0.001	23 (100)	51 (96.2)	1.000
Arteriolosclerosis, *n* (%)	17 (100.0)	0	18 (100.0)	<0.001	3 (18.8) ‡	7 (58.3) ‡	0.050
Total CMB number, median (IQR)	7 (3–140) **	161 (42.5–198) **	0 (0–3) **	0.015	161 (43.5–198) #	11 (5.5–140) #	0.465
CMI number, median (IQR)	27 (5–?)†	31 (1.5–104)†	0 (0–0)†	0.180	47.5 (10–124) ||	27 (5–?)||	0.480

*Eighteen and 49 patients in non-severe and severe group had data on hypertension, respectively

^†^Three, 5, and 2 patients in CAA + arteriolosclerosis, strict CAA, and strict arteriolosclerosis group had data on CMI number, respectively

^‡^Sixteen and 12 patients in non**-**severe and severe group had data on arteriolosclerosis, respectively

^§^Twelve, 30, and 16 patients in CAA + arteriolosclerosis, strict CAA, and strict arteriolosclerosis group had data on hypertension, respectively

^||^Four and 3 patients in non-severe and severe group had data on CMI number, respectively

^#^Both severe and non-severe groups had 5 patients with data on total CMB number

**Five, 5, and 11 patients in CAA + arteriolosclerosis, strict CAA, and strict arteriolosclerosis group had data on CMB number, respectively

For patients with ICH due to strict arteriolosclerosis, the majority (61.1%) of ICH were located in deep regions of the brain. The presence of arteriolosclerosis was significantly associated with hypertension (percentage of hypertension in arteriosclerosis vs. non-arteriolosclerosis: 74.6 vs. 36.4%, *p*=0.001). This association remained significant after correcting for age and sex (adjusted OR 3.329, 95% CI 1.056–10.495, *p*=0.040).

### The Correlation Between MRI and Pathological Changes of CSVD Markers in Primary ICH

#### White Matter Changes

Overall, six studies [15, 18, 20, 30, 32, 36] investigated white matter changes on CT or MRI. Of these, three studies [18, 20, 30] examined the correlation between MRI and pathological changes in white matter lesions (Table 3). White matter changes were associated with rarefaction of myelin staining, diffuse or patchy myelin loss, diffuse white matter edema, and even advanced white matter loss [20, 30]. Histologically, the white matter appeared vacuolated and was accompanied by swollen oligodendrocytes and astrocytic proliferation [18, 30]. Amyloid plaques that were positive for Aβ were occasionally observed in the white matter [18]. However, CAA-associated vasculopathy did not always result in ischemic white matter lesions [18].

**Table 3:** Neuropathological changes of white matter changes on imaging

Ref.	Macroscopic changes of WMH	Associated cell changes		Aβ-positive blood vessels in the white matter	Amyloid plaques in the white matter
		Cell changes	Location		
Fazekas 1999 [20]	Correspond to rarefaction of myelin staining and diffuse white matter edema	Unclear	Unclear	Unclear	Unclear
Gray 1985 [30]	Diffuse or patchy myelin loss	Vacuolated white matter, swollen oligodendrocytes, and astrocytic proliferation.	Centrum semiovale, and parietooccipital regions	Unclear	Unclear
Oide 2003 [18]	Myelin pallor, present in 1/12 of the autopsied cases	Focal astrogliosis, present in 1/12 of the autopsied cases	Subcortical white matter	A small number of vessels, without wall-thickening	Occasionally observed in 1 autopsied case

*WMHs* white matter hyperintensities, *Aβ* amyloid beta

#### Cerebral Microbleed

Twelve studies [13, 15–17, 20, 22, 25, 31, 32, 34, 35, 37] investigated CMB on MRI, and nine [13, 16, 17, 20, 22, 25, 31, 34, 35] of them retrieved CMB for histopathological analysis. The pathological changes of CMB in primary ICH are presented in Table 4. There existed inconsistencies in CAA severity around a CMB [13, 25]. The vessels involved in CMB were enlarged and fibrinoid necrotic, with extensive remodeling of the vessel wall [13]. On the other hand, a high CMB count was associated with an increased wall thickness of amyloid-positive vessels, and this increased thickness of vessel walls might be more prone to CMB formation than macrobleed formation [34].

**Table 4:** Neuropathological changes of other CSVD markers in ICH patients

Study	Main pathological findings
CMB	
Fazekas 1999 [20]	Twenty-one of 34 CMBs on MRI were confirmed pathologically, characterized by focal accumulation of hemosiderin-laden macrophages, and sometimes tissue necrosis. Hemosiderin deposits (often smaller and contained lesser hemosiderin-laden macrophages) were observed in the brains which were negative for CMB on MRI.
Hernandez-Guillamon 2012 [22]	Fifteen chronic bleeds containing hemosiderin or other iron storage complexes were found in the lobes, among which 7 were positive for Aβ staining.
van Veluw 2019 [13]	The rupture site of vessel contained less Aβ, less intact smooth muscle cells, and extensive fibrin. The upstream or downstream of the rupture site contained more Aβ. The involved vessels of CMB were enlarged and fibrinoid necrotic, with extensive vessel wall remodeling.
Guidoux 2018 [35]	Forty-five of 48 CMBs on MRI were confirmed pathologically, corresponding to small bleeds with lytic red bloods cells for recent CMB, and macrophages for old CMB. There was no significant difference in CMB size on pathology between those with and without CAA.
Greenberg 2009 [34]	Patients with high CMB count (>50) had increased thickness of amyloid-positive vessel wall than those with few CMBs (<3).
Shelton 2015 [31]	No CMB on neuroimaging. Autopsy showed hemosiderin deposition with spongiosis in perivascular regions.
Jolink 2022 [25]	Total CMB number was higher in lobar ICH than non-lobar ICH, without reaching a significant difference. Cortical CMB were more frequently noted in superficial layers of cortex in lobar ICH, and in deep layers of cortex in non-lobar ICH. CAA was severer surround the CMB on lobar ICH than that in non-lobar ICH. Severe CAA of the involved vessels of CMB was infrequently observed.
van Veluw 2016 [17]	Ex vivo MRI detected 171 CMBs (all located in cortical ribbon), compared to ~66 in vivo MRI. Acute CMBs were characterized by accumulation of intact red blood cells. Old or subacute CMBs corresponded focal hemosiderin deposits.
Schrag 2010 [16]	Seven, 2, and 2 CMBs in cortical grey matter, deep grey matter, and white matter, respectively. Susceptibility-weighted imaging overestimated CMB size.
CMI	
Ter Telgte 2020 [12]	Acute cortical CMI: tissue pallor consisting of eosinophilic neurons, absent or mild reactive astrocytes, and absent or fragmented microglia. Chronic cortical CMI: tissue loss or central cavitation, with many reactive astrocytes and few reactive or amoeboid microglia.
van Veluw 2019 [13]	Many Aβ-positive vessels surrounding a CMI. Mild fibrin and absent or only few intact smooth muscle cells in the vessels involved in CMI; the involved vessels were intact but stiff due to the deposition of Aβ, with relatively narrow lumens.
Jolink 2022 [25]	More CMI in lobar ICH than non-lobar ICH. Cortical CMI were more frequently noted in superficial layers of the cortex in lobar ICH, and in deep layers of the cortex in non-lobar ICH. CAA was severer surround the CMI in lobar ICH than that in non-lobar ICH. Severe CAA of the involved vessels of CMI was infrequently observed.
van Veluw 2016 [17]	CMI tended to cluster, characterized by ischemic or shrunken neurons for acute CMI and tissue loss and gliosis for chronic CMI.
Lacunes	
Fazekas 1999 [20]	Five of 7 patients with MRI lacunes in the basal ganglia and thalami were histologically confirmed.
Atrophy	
Poyuran 2019 [15]	Parenchymal β-amyloid plaques, in addition to vascular Aβ deposition, were noted in cases with diffuse cerebral atrophy.
CSVD burden	
Charidimou 2016 [36]	Higher CSVD score was associated with CAA-related abnormities on pathology and the presentation of ICH.
Pasi 2020 [26]	Five of 8 patients could be rated for CSVD score. Deep ICH were scored 1 for moderate arteriosclerosis, and 3 or 4 for mild arteriosclerosis. Lobar ICH without arteriosclerosis were scored 2.

*CSVD*, cerebral small vessel disease; *CAA*, cerebral amyloid angiopathy; *CMB*, cerebral microbleed; *ICH*, intracerebral hemorrhage; *MRI*, magnetic resonance imaging; *WMHs*, white matter hyperintensities; *CMI*, cerebral microinfarct

#### Cerebral Microinfarct

Four studies [12, 13, 17, 25] investigated CMI, and all four investigated pathological changes of CMI, as detailed in Table 4. The findings suggested that small DWI lesions could be matched to acute CMI histopathologically, characterized by tissue pallor consisting of eosinophilic neurons, absent or only mild reactive astrocytes, and absent or fragmented microglia. And chronic cortical CMI was associated with tissue loss or central cavitation, as well as many reactive astrocytes around the lesions and few reactive or amoeboid microglia [12]. CAA was found to be severe surrounding a CMI, particularly for CMI in those with lobar ICH.

#### Lacunes, EPVS, and Brain Atrophy

Each of the three imaging markers was investigated in a separate study (Table 1). Patients with brain atrophy corresponded to amyloid deposition in the parenchyma and small vessels [15]. However, the pathological changes of lacune and EPVS in primary ICH were not investigated.

#### Total CSVD Burden

Two studies [26, 36] investigated the total CSVD burden (Table 4). A direct histopathological-MRI study demonstrated that the total CAA burden of CSVD, as determined by summing the four most characteristic MRI markers of CAA, was associated with the presentation of symptomatic CAA-related ICH (versus CAA without ICH) and CAA-related microangiopathy on pathology [36].

## Discussion

We presented here a comprehensive systematic review of published articles that investigated ICH with pathology-proven evidence for arteriolosclerosis and CAA. Our study directly compared MRI and pathology to clarify the pathological changes of CSVD imaging markers. To the best of our knowledge, this is the first review to assess the interaction between arteriolosclerosis and CAA by summarizing published cases with a pathology diagnosis of CAA or arteriolosclerosis in ICH. Our findings provide pathological evidence that there might be differences in lobar ICH and total microbleed number among CAA + arteriolosclerosis, strict CAA, and strict arteriolosclerosis, and there might be an association between arteriolosclerosis and severe CAA, which needs to be investigated further in future studies.

Although the SVWCs in CAA-ICH were found to be similar to those in CAA without ICH, in terms of the location of Aβ deposition and the changes in the lumen [40], our findings suggest that severe CAA (usually characterized by double barreling and/or fibrinoid necrosis) is highly prevalent in CAA-ICH. Our results, together with the findings of Vonsattel et al. [21], indicate that a severe degree of CAA represents an important feature of CAA-ICH [21].

Regarding the CSVD markers in ICH, we found that patients with CAA had higher number of total CMB, particularly for patients with strict CAA, as compared to those without CAA on pathology. This finding was in line with previous study by Edip Gurol et al. that found that patients with CAA-ICH had higher number of total CMB as compared to those with non-CAA related ICH [41]. In addition, the total CAA burden assessed in neuroimaging was associated with the presentation of symptomatic CAA-related ICH and CAA-related microangiopathy on pathology. Therefore, it might be reasonable to speculate that higher individual CSVD markers indicate greater Aβ severity. However, our analysis of individual patient data showed that severe CAA was not associated with CMB number, which is in line with the findings of another study that found CMB to be frequently located around Aβ negative small vasculature [42]. In addition, one amyloid positron emission tomography study evaluating CAA burden in both the ICH-affected hemisphere and the ICH-free hemisphere indicated that amyloid burden was similarly distributed and ICH was unlikely to be directly linked to amyloid burden [43]. Taken together, these findings challenge the hypothesis that the ruptured vessel reflects a significant Aβ load [42].

As expected, hypertension was significantly associated with arteriolosclerosis, which was highly prevalent in CAA-ICH as well. It is widely accepted that lobar hemorrhage is required for CAA diagnosis per the Boston Criteria [44]. In this study, we found that all cases of strict CAA-ICH were located in the lobes. However, we also found that in some patients with the coexistence of CAA and arteriolosclerosis on pathology, the hemorrhage could be located in deep territories, suggesting that the presence of deep ICH could not preclude the possibility of amyloid angiopathy in the elderly patients.

Overall, the pathology of CSVD markers in ICH was under-researched, with most studies focusing on CAA-ICH. While the majority of CMBs observed on MRI could be confirmed histopathologically, several CMBs could not be confirmed, with no abnormality or small cavities at neuropathological amination [20, 35]. Compared with standard histopathological sections, MRI tends to underestimate the total burden of CMB [20], as well as CMI [13]. The lesions that could not be retrieved for histological analysis were generally too small [25]. Therefore, future studies with high-field MRI scanners are warranted to capture the total burden of these two markers [20, 35]. Of note, CMB on MRI was traditionally recognized to represent foci of past hemorrhage [20, 22]. However, this review found that CMB could also correspond to recent microhemorrhage with small bleeds in patients with ICH [35].

Another interesting finding was the inconsistency in CAA severity around CMB [13, 25]. This inconsistency might be attributed to the different methodologies used by the two studies. Jolink et al. [25] investigated CAA severity for each patient by taking samples with multiple lesions, while van Veluw et al. [13] assessed CAA for each section that contained CMB. Studies that directly correlated MRI and pathology of lacunes, EPVS, and atrophy were scarce. Thus, future studies are warranted to further elucidate the CAA pathology around CMB and CMI, and to assess the pathology of lacunes, EPVS, and atrophy in ICH.

### Strength and Limitation

The present systematic review provided the most recent and comprehensive overview of the pathology of CSVD in primary ICH. Studies were comprehensively searched from databases using a systematic search strategy, as well as from handsearching of references of relevant studies. However, the majority of included studies only assessed the presence of CAA on pathology, without information on arteriolosclerosis, resulting in a relatively small number of cases with CAA + arteriolosclerosis, strict CAA, and strict arteriolosclerosis. Despite this, our study has the largest number of ICH cases with pathology-proven etiology across the literature and firstly provides the clinical and imaging information for CAA+arteriolosclerosis, strict CAA, and strict arteriolosclerosis.

### Conclusion and Considerations for Future Research

In summary, our study provides synthesized pathological evidence for the hypothesis that there might be an interaction between CAA and arteriolosclerosis. Future studies are needed to verify our findings. The pathological changes of CSVD features in neuroimaging are heterogeneous, and MRI-histopathological correlation studies of lacunes and EPVS remain scarce. Therefore, further investigation is needed to explore the pathological changes of CSVD markers by ICH etiology. Additionally, studies using advanced MRI techniques with a high spatial resolution to visualize structural and functional brain changes of CSVD in ICH are also warranted.

### Supplementary Information

ESM 1 (DOCX 27 kb)
