# Supplementary material for: Pathological Changes of Small Vessel Disease in Intracerebral Hemorrhage: a Systematic Review and Meta-analysis
Source: Transl Stroke Res. 2023 Jun 6;15(3):533–44. doi: 10.1007/s12975-023-01154-4 (PMC11106194; doi:10.1007/s12975-023-01154-4)
Supplement: Supplementary file 1 — (DOCX 27 kb) [file 12975_2023_1154_MOESM1_ESM.docx]

**Supplementary table Neuropathological SVWCs in CAA-ICH patients**

| **Study** | **Diagnostic method of CAA** | **CAA-related** **SVWCs** | | **Arteriolosclerosis** | **Senile plaques** | **Blood vessel changes in/ around hematoma** |
| --- | --- | --- | --- | --- | --- | --- |
|  |  | **Location** | **Vessel wall changes** |  |  |  |
| Doden 2016 [32] | Congo red stain; immunohistochemis-try against Aβ | Mainly in cortical and meningeal  tissue, and arachnoidal tissue occasionally | Unclear | Unclear | Unclear | Aβ-positive blood vessels in the hematoma |
| Fazekas 1999 [20] | Congo red staining | Unclear | Moderate and severe CAA each, which associated with remote foci blood leakage | Moderate lipofibrohyalinosis | Unclear | Unclear |
| Gilbert 1983 [33] | Congo red stain | Leptomeninges, cortex, and cerebellum | Thickness, stenosis or occlusion small arteries with hyaline material, some associated with underlying cerebral infarction | Present in 2 patients | Abundant in 10 patients | Brain tissues adjacent to hematoma contained severe CAA |
| Gray 1985 [30] | Congo red staining | Leptomeninges  and CC | FN and hyalinous change | Diffuse arteriosclerosis | Severe and widespread in 5 and discrete and confined in 4 patients | Unclear |
| Hernandez-Guillamon 2012 [22] | Immunohistochemis-try against Aβ | Cerebral parenchyma | CAA grade 1-4; occasional microaneurysms; MMP-2 expressed in Aβ-positive vessels far from hematoma † | Unclear | Unclear | Unclear |
| Ishii 1984 [19] | Congo red stain | Mainly in leptomeninges and CC, and also in basal ganglia, thalamus, and cerebellar to a lesser extent. | Moderate CAA in 4 patients, and severe in 3 patients. | Present in 3 patients | Present in all patients | Unclear |
| Lin 2018 [14] | Immunohistochemis-try against Aβ | Cortex over the hematoma | Three, 2, 3, and 16 patients had CAA grade 1, 2,3 and 4, respectively. † | Unclear | Unclear | Unclear |
| Oide 2003 [18] | Congo red staining; immunohistochemis-try against tau, glial fibrillary acidic protein, Aβ40, and Aβ42 | Leptomeninges, CC, and occasionally WM | Obliterative, Onion-like thick with amyloid infiltration, double barreling; splitting of internal elastic lamina and intimal fibrosis. CAA severity 2+~3+ in grey matter. | Present in 2 of the 12 autopsied cases | Present in 78.1% of patients (more strongly positive for Aβ-42) | Unclear |
| Poyuran 2019 [15] | Congo red staining; immunohistochemis-try against Aβ | Leptomeninges  and superficial cortex | FN, RBC leakage and amyloid leakage following rupture, microaneurysms and double barreling. CAA grade 2-4. † | Unclear | Aβ plaques in parenchyma | Unclear |
| Schrag 2010 [16] | Immunohistochemis-try against Aβ | Unclear | CAA grade 3. † | Unclear | Unclear | Unclear |
| Shelton 2015 [31] | Congo red stain; immunohistochemis-try against Aβ | CC | Thick and hyalinized blood vessels | Unclear | Present | Microangiopathic changes, hyalinosis, and fibrinoid necrosis of the vessel walls adjacent to hematoma |
| Takeda 2012 [29] | Immunohistochemis-try against Aβ | SAS, CC, and ISH | A few double-barreled vessels, and occasionally fibrosis | Unclear | Unclear | Mainly arteries and few veins ruptured; fibrosis and fibrin formation in the hematoma |
| Takeda 2018 [24] | Immunohistochemis-try against Aβ | Meninges, CC, intrasulcal hematoma, and SAS | Thick, fuzzy, FN, micro-aneurysmal dilatation, and hyalinous onion-like change | Mild | Obvious in the CC, and spotty distribution | Ruptured  Aβ-positive blood vessels in the hematoma |
| van Etten 2014 [38] | Unclear | Unclear | Moderate to severe CAA in all patients | Unclear | Unclear | Unclear |
| van Veluw 2016 [17] | Immunohistochemis-try against Aβ | Unclear | Severe CAA: grade 4 † | Moderate | Unclear | Unclear |
| Vonsattel 1991 [21] | Congo red staining | Leptomeninges  and parenchyma | Moderate and severe CAA in 2 and 15 patients, respectively. FN in 12 patients. | Unclear | Neuritic plaques in 14 patients. | Unclear |
| Yoshimura 1992 [39] | Congo red staining and ultrastructural demonstration of amyloid fibrils | Meningocortical regions of cerebrum and cerebellum, and ocasionionally in the hippocampus, as well as the moter and visual cortices | Massive amyloid fibrils in the tunica adventitia and media; some “double-barrelling” and microaneurysms with amyloid. | Unclear | Present in all patients | Unclear |

*The original study included 12 CAA patients (aged 55-83 years) and 9 of the 12 patients has hematoma.

† CAA grade by Greenberg and Vonsattel.

**Abbreviation**: SVWCs, small vessel wall changes; CAA: cerebral amyloid angiopathy; ICH: intracerebral hemorrhage; SAS: subarachnoid space; CC: cerebral cortex; ISH: intrasulcal hematoma; RBC, red blood cell; WM: white matter; FN: fibrinoid necrosis; Aβ: Amyloid beta.
